# Network Pharmacology-Based and Experimental Identification of the Effects of Paeoniflorin on Major Depressive Disorder

**DOI:** 10.3389/fphar.2021.793012

**Published:** 2022-02-02

**Authors:** Sha Zhang, Mingchen Jiang, Shuxia Yan, Miaomiao Liang, Wei Wang, Bin Yuan, Qiuyue Xu

**Affiliations:** ^1^ School of Nursing, Nanjing University of Chinese Medicine, Nanjing, China; ^2^ Department of Pediatrics, Affiliate Hospital of Nanjing University of Chinese Medicine, Nanjing, China; ^3^ School of Medicine & Holistic Integrative Medicine, Nanjing University of Chinese Medicine, Nanjing, China

**Keywords:** major depressive disorder, paeoniflorin, network pharmacology, experimental verification, treatment targets

## Abstract

**Objective:** Major depressive disorder (MDD) is one of the most common psychiatric disorders, the diagnosis and treatment of MDD are major clinical issues. However, there is a lack of effective biomarkers and drugs diagnosis and therapeutics of MDD. In the present study, bioinformatics analysis combined with an experimental verification strategy was used to identify biomarkers and paeoniflorin targets for MDD diagnosis and treatment.

**Methods:** Based on network pharmacology, we obtained potential targets and pathways of paeoniflorin as an antidepressant through multiple databases. We then constructed a protein-protein interaction network and performed enrichment analyses. According to the results, we performed *in vivo* and *in vitro* experimental validation.

**Results:** The results showed that paeoniflorin may exert an antidepressant effect by regulating cell inflammation, synaptic function, NF-κB signaling pathway, and intestinal inflammation.

**Conclusion:** NPM1, HSPA8, HSPA5, HNRNPU, and TNF are the targets of paeoniflorin treatment. In addition, we demonstrated that paeoniflorin inhibits inflammatory cytokine production *via* the p38MAPK/NF-κB pathway and has neuroprotective effects on the synaptic structure. Our findings provide valuable evidence for the diagnosis and treatment of MDD.

## Introduction

Major depressive disorder (MDD) is characterized by changes in affective and cerebral neurophysiological functions, with episodes lasting for at least 2 weeks. MDD has a lifetime prevalence of 13.2% and an annual depression prevalence of 5.3% per individual and is one of the leading causes of disability (Olfson et al., 2017). The prevalence of mental health disorders has continued to increase in recent years, and nearly 3.5% of the world’s population experienced depression in 2017. At present, the clinical diagnosis of depression is often mainly based on patient complaints, clinical observations, the Hamilton Depression Scale (HAMD), and auxiliary laboratory tests (Galvão et al., 2021). Antidepressants remain the mainstay of treatment for major depressive symptoms; however, there are still some undesirable disadvantages, such as limited efficacy, uncertain safety, low response rate, and serious adverse effects.

Compared with Western medicine, traditional Chinese medicine (TCM) has the advantages of multiple targets and fewer side effects in the treatment of depression. Classical prescriptions such as Xiaoyao powder (Hou et al., 2020), Sini powder (Cai et al., 2021), and Chaihu Shugan powder (Yan et al., 2020) have all achieved good antidepressant effects in the clinic. Radix Paeoniae Alba is a major component of Chaihu Shugan powder and Sini powder. Paeoniflorin is a monoterpenoid compound found in Radix Paeoniae Alba and has many biological activities. Paeoniflorin has been reported to exhibit antidepressant-like effects in several animal models of depression and exerts a neuroprotective effect (Wang et al., 2021).

**FIGURE 1:**
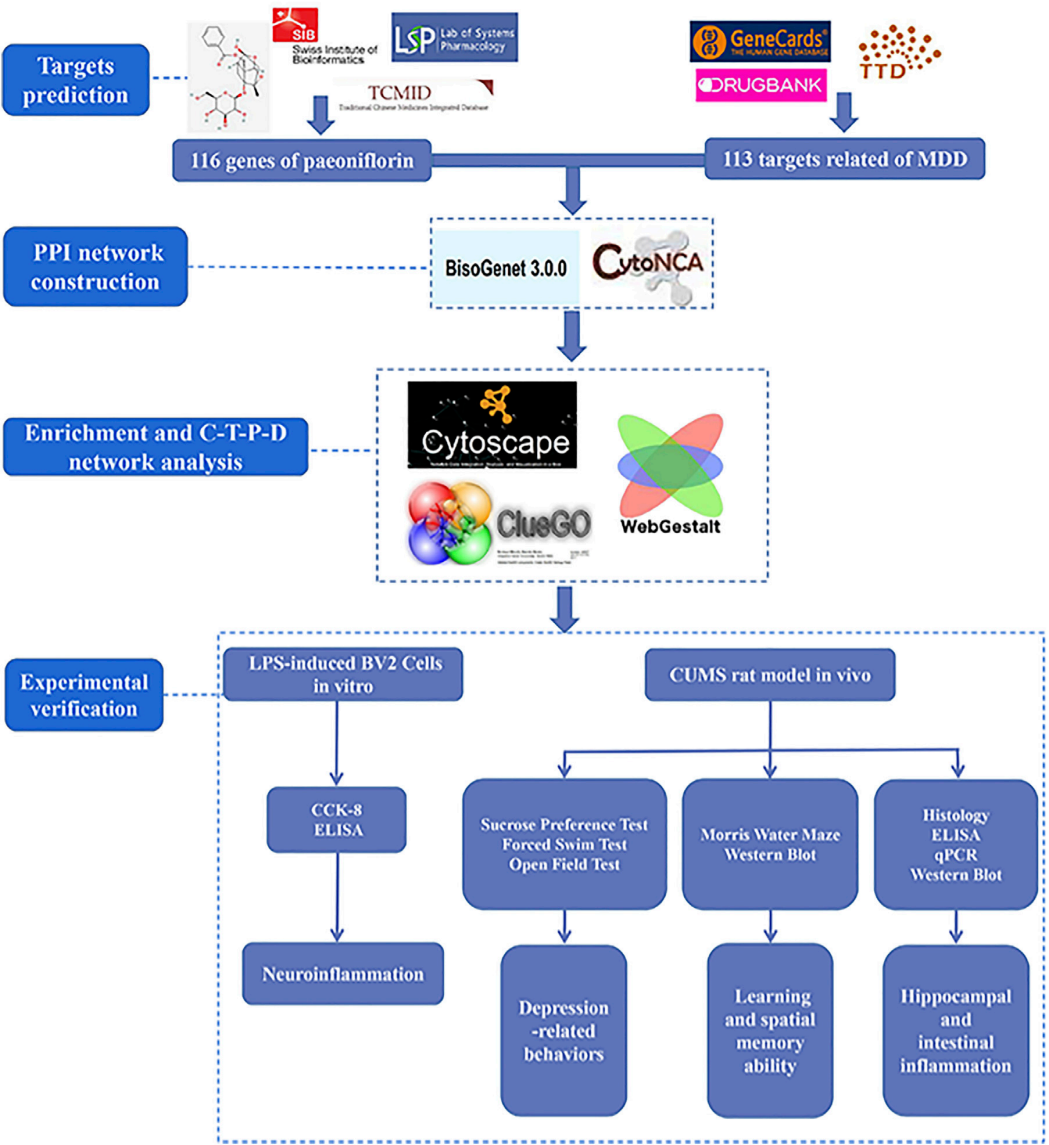
Technical strategy of the current study.

TCM compounds consist of many small molecular compounds that can bind to a variety of proteins, sometimes instantaneously or simultaneously at multiple sites (Bai et al., 2013; Ohlson, 2008) through the complex synergistic or antagonistic interaction between the components of Chinese medicine combinations, targeted in the treatment of diseases to increase efficacy and reduce toxic side effects. Network pharmacology is a pharmacological branch of systems biology, bioinformatics, and high-throughput omics analysis (Zhou et al., 2020). The concept of “holistic view” displayed by network pharmacology conforms to the multi-component, multi-target, and multi-pathway intervention mechanism of TCM. TCM network pharmacology was derived from the combination of TCM and network pharmacology (Li and Zhang, 2013). Network node targets were used to establish various associations between small molecules of TCM and diseases, and network characteristics generated by network topology and dynamics expressed the combination rules and overall regulatory effects of TCM (Li et al., 2011). This “network target” can effectively decipher the molecular mechanism of multiple therapeutic effects of TCM and determine its effective components or combination (Li et al., 2011; Li and Zhang, 2013). Hence, TCM network pharmacology can be used as a scientific basis for the systematic understanding of TCM at the molecular level. Therefore, in this study, network pharmacology was used to explore the targets and related pathways of paeoniflorin in the treatment of MDD. Based on this, paeoniflorin was validated for its pharmacodynamic effects on microglia (Cheng et al., 2021) and the chronic unpredictable mild stress (CUMS) model (Chen et al., 2019), in order to explore the mechanism of paeoniflorin in the treatment of MDD. Our study provides a more specific and effective way of offering new insights into the mechanisms of paeoniflorin in the treatment of MDD. The technical strategy of the current study is shown in Figure 1.

## Materials and Methods

### Paeoniflorin Target Prediction and MDD-Related Target Screening

The compound structure of paeoniflorin downloaded from PubChem (Kim et al., 2021) website was uploaded to SwissTargetPrediction database (Gfeller et al., 2014), Traditional Chinese Medicine Systems Pharmacology (TCMSP) database (Ru et al., 2014) and Traditional Chinese Medicine Integrative database (TCMID) (Huang et al., 2018) while its parameters were set as default values. The MDD-related targets were obtained from the Therapeutic target database (TTD) (Wang et al., 2020), Drugbank (Wishart et al., 2018), and GeneCards (Stelzer et al., 2016) database. Finally, targets screened were normalized by UniProt database (UniProt Consortium., 2019).

### Protein–Protein Interaction Network Analysis

The BisoGenet plug-in of Cytoscape (Martin et al., 2010) was applied to establish the protein-protein interaction (PPI) network between paeoniflorin and the relevant gene of MDD. The intersection network of two PPI networks was extracted by using “Merge” function in Cytoscape, and then the attribute values of each node in the intersection network were analyzed by using CytoNCA plug-in (Tang et al., 2015). Then, the nodes whose degree value is more than two times of the median were screened to be “Hit hubs.” At last, each node of the Hit hubs network was calculated the properties and selected by meeting greater than degree, closeness, betweenness, neighborhood connectivity and local average connectivity at the same time as the key targets.

### Functional Enrichment Analysis of Targets and Compound-Target-Pathway-Diseases Network Construction

The key targets were uploaded to the CluoGO plug-in of Cytoscape (Bindea et al., 2009) to conduct gene ontology (GO) enrichment analysis, which includes biological process (BP), molecular function (MF), and cellular component (CC) analysis. Afterwards, the key targets carried out Kyoto Encyclopedia of Genes and Genomes (KEGG) enrichment analysis by WEB-based GEne SeT AnaLysis Toolkit seleting species “*Homo sapiens*,” and screened the possible pathway of paeoniflorin treating MDD (Wang et al., 2013). The Requary filter conditions of both GO and KEGG enrichment analyses were FDR <0.05. After enrichment analysis of targets and pathways, we constructed the compound-target-pathway-disease network (C-T-P-D network) to visualize their relationship.

### Chemical Reagents

Paeoniflorin was purchased from Shanghai Yuanye Bio-Technology Co., Ltd. (Shanghai, China) (Lot. Number: L07M9Q60553), dissolved and diluted in the dulbecco’s modified eagle medium (DMEM). Fluoxetine was purchased from Jiangsu Provincial Hospital of Traditional Chinese (Medicine Manufacturer: Patheon France, National Medicine Standard J20180022, Specification: 20 mg). Fetal bovine serum (FBS) was obtained from Gibco. DMEM and trypsin were purchased from Hyclone. NF-κB p65, IκBα, p38MAPK and anti-rabbit IgG horseradish conjugate secondary antibody were purchased from Cell Signaling Technology. PSD95 and BDNF were procured from Proteintech Group, Inc., (Wuhan, China). Primers used in qPCR were designed and synthesized by Sangon Biotech (Table 1). FastPure® Cell/Tissue Total RNA Isolation Kit V2, HiScript III RT SuperMix for qPCR (+gDNA wiper) and ChamQ Universal SYBR qPCR Master Mix was purchased from Vazyme (Nanjing, China). IL-6, TNF-α and IL-1β Elisa Kit was purchased from Solarbio. CCK-8 kit was purchased from Beyotime (Nanjing, China).

**TABLE 1:** Scoring for Jejunal inflamation.

Severity of inflammatory cell infiltrate	Score
N/A	0
Minimal	1
Mild	2
Moderate	3
Marked	4

### Cell Treatments and Viability Assay

BV2 cells were suspended in DMEM supplemented with 10% FBS at 37°C with 5% CO_2_. Lipopolysaccharide (LPS) concentration gradients were designed as 0, 0.05, 0.1, 0.5, 1, 2.5, 5, 7.5, 10 and 20 μg/ml, then detected the effect of LPS on cell activity by CCK-8 kits. BV2 cells was exposed to paeoniflorin in concentrations of 0–1000 μM for 0.5–48 h, and CCK-8 detection was performed when reaching the point of time to measure the effect of paeoniflorin on cell activity. Each well of the plate was added into 10 μL of CCK-8 solution, and incubated for 4 h at 37°C with 5% CO_2_. The optical density at 450 nm was measured on a microplate analyzer for data analysis.

### Animals and Grouping

Healthy male adult Sprague Dawley rats (weight 200–210 g), SPF grade (Certificate No. 201906447), were purchased from Nanjing Qinglongshan animal Co., Ltd [No. scxk (Su) 2018-0049, China]. After arriving, they were reared for 7 days to adapt to the environment in where room temperature was 21.3–25.2°C, humidity 55 ± 4%, free to eat and drink and natural light. The rats were weighed weekly. After 7  days of acclimatization, male SD rats were randomly divided into five groups (*n* = 8 per group): Control group, Control + Pae (20 mg/kg) group, CUMS group, CUMS + Flu (10 mg/kg) group and CUMS + Pae (20 mg/kg) group. The doses of paeoniflorin and fluoxetine were selected on the basis of available scientific reports (Fernandes and Gupta, 2019; Huang et al., 2015; Liu et al., 2019; Zu et al., 2017).

### CUMS Rat Model and Dosing

In order to ensure the unpredictability of the stressors, all stressors were randomly scheduled and presented not more than five times during modeling. The specific operation method is as follows: 1) 24-h wet cages, 2) 5-min tail pinch, 3) 5-min cold swim, 4) 4-h physical restraint, 5) 24-h food deprivation, 6) 24-h water deprivation, 7) Day and night reversed, 12 h lights on (12 h lights off), 8) 12-h cage tilt (45° from the horizontal). The stimulation schedule was as follows: week 1: (2) (3) (1) (6) (8) (5) (4); week 2: (7) (6) (4) (2) (3) (7) (6); week 3: (8) (4) (3) (1) (7) (2) (5); week 4: (6) (8) (4) (2) (3) (7) (1); week 5: (4) (8) (9) (2) (6) (5) (3).

**FIGURE 2:**
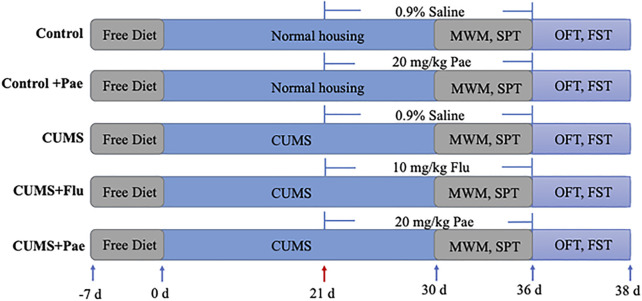
Schedule of dosing.

The establishment of CUMS model lasted 5 weeks. The drugs dissolved in 0.9% saline were given in the last 2 weeks and stored in refrigerator at 4°C.Each rat was intragastrically administered with a volume of 10 ml/kg at the same time each day. The groups received CUMS stimulation after 30 min of lavage for 35 days except the Control group and the Control + Pae group. The administration was started on the 21st day and lasted for 2 weeks, afterwards various behavioral tests were conducted (Figure 2).

### Behavioral Tests

Sucrose Preference Test (SPT): According to the published literature for reference, the rats in each group were given adaptive sugar drinking training before the experiment. In the adaptive training, rats in each group were given two bottles of 1% sucrose water for 1 day, then one bottle of sugar water was changed into pure water for another day. After 24-h water and food deprivation, SPT started: rats in each cage were given one bottle of 1% sucrose water weighed as W1 and one bottle of pure water weighed as W2. One hour later, we took down the kettle of each cage, weighed the consumption recorded as W3 and W4 respectively, thus SPT = (W1−W3)/(W1+W2−W3−W4) ×100%. SPT schedule: the 0th, second, fourth and fifth weeks. In order to decrease the experimental error, ensuring that the time period and feeding environment of each experiment were consistent.

Forced Swim Test (FST): FST was conducted between 9 am and 12 am. After 30 min of gavage, rats in each group were separately placed in a plastic bucket (50 cm in height and 25 cm in diameter) filled water (30 cm in depth) at 25°C for 5 min. The immobility time of rats in each group was automatically recorded and analyzed by the Depression Scan software. At the end of the experiment, the rats were dried and put back into the cage.

Open Field Test (OFT): OFT was performed by a custom-made open field (50 cm × 50 cm × 50 cm), in the center of which the rats were placed. After the experiment, the data were analyzed and the movement distance of each rat was calculated. Keep the environment quiet once the test started.

Morris water maze (MWM): The MWM experiment lasted for 6 days consecutively, and the hidden platform experiment needed to conduct from first to fifth day. Firstly, the bucket was divided into four quadrants and the rats were put into water from different quadrants every day. The training duration is 120 s while the training interval of each quadrant is 30 s, and we recorded the changes of latency of rats at this time. In order to prevent the influence of different water entry distance and fixed sequence quadrant water entry on the experimental results, we chose the starting positions semi randomly in four quadrants. Besides we tried to keep the rats face the barrel wall when entering the water, while keeping the same distance and water entry sequence. Space exploration experiment was performed on the sixth day in which the platform should be removed and rats were put into water from the fixed quadrant (the farthest quadrant of the platform). The number of times crossing the platform and the latency time in 120 s were counted to evaluate the learning and spatial memory ability of the rats. Keep the environment quiet once the test started. After the experiment, we dried the rats with towel and put them back the cage.

### Sample Preparation

The rats anesthetized by isoflurane gas were bled from the abdominal aorta. Blood was collected and centrifuged in tubes with heparin anticoagulant to obtain supernatant. Following cervical dislocation, the whole brains were harvested, and hippocampi were dissected from it. Sections (5–8 cm) of jejunum were collected and half of which fixed in 4% paraformaldehyde fixative solution for making pathological slices. *Hippocampus* and the rest of the jejunum were flushed with PBS, blotted with filter papers and stored at −80°C ultimately. To prevent protein degradation, all steps were performed on ice.

### ELISA

The expression level of IL-6, TNF-α, and IL-1β in hippocampus tissues and treated BV2 cells were measured by the ELISA Kits, the operations were conducted strictly according to the kit instructions.

### Western Blot

Proteins were extracted from tissues using RIPA buffer supplemented with protease inhibitors. Protein concentration was quantified by BCA assay refer to the instructions. The protein was separated *via* 12 or 15% SDS polyacrylamide gel electrophoresis according to molecular weight and transferred to PVDF membrane. Membranes was blocked *via* 5% skimmed milk at room temperature for 1 h and washed with TBST thrice. Then incubation with primary antibody was accomplished overnight at 4°C and second antibody was incubated for 1 h at room temperature. After washing three times with TBST, the immunoreactive bands were detected by ECL detection reagents and visualized by image lab software.

### Histology

Dehydrated jejunal tissues were stained with hematoxylin and eosin solution for 5 min. The slices were put into anhydrous ethanol I, anhydrous ethanol II, anhydrous ethanol III and xylene I for 5 min in turn for dehydration and sealing. In particular, the presence of inflammatory infiltration and epithelial abnormalities were evaluated at ×200 magnification, the scoring criteria are shown in Table 1.

### qPCR

The total RNA was extracted from the jejunal tissues by FastPure® Cell/Tissue Total RNA Isolation Kit and transcribed into cDNA by HiScript III RT SuperMix for qPCR (+gDNA wiper). Then ChamQ Universal SYBR qPCR Master Mix was used to perform qPCR in a volume of 10 μL. The expression levels in genes were quantifified by Quant Studio 7 Flex PCR system. Applying 2^−ΔΔCt^ method to calculate and analyse the data, and GAPDH was used as reference gene for standardization. The primer sequences of genes relatived with MDD were as follows (forward and reverse) (Table 2).

**TABLE 2:** Relatived gene primer sequences.

Gene	Forward prime (5′- 3′)	Reverse prime (5′- 3′)
IL-1β	GCA​ACT​GTT​CCT​GAA​CTC​AAC​T	ATC​TTT​TGG​GGT​CCG​TCA​ACT
IL-6	CTG​CTC​TGG​TCT​TCT​GGA​GT	GGT​CTT​GGT​CCT​TAG​CCA​CT
TNF-α	GGA​ACA​GTC​TGG​GAA​GCT​CT	GGA​ACA​GTC​TGG​GAA​GCT​CT
β-actin	GGA​ACA​GTC​TGG​GAA​GCT​CT	CAC​ACA​GAG​TAC​TTG​CGC​TC

### Statistical Analysis

GraphPad Prism 8.0 software was employed to conduct statistical analysis and plot. All data were presented as mean ± SEM. The significance of each group was verified with one way or two ways ANOVA, and differences in multiple groups were analyzed by Dunnett’s post hoc. A *p* value <0.05 was considered statistically significant.

## Results

### Target Screening and Protein-Protein Interaction Network Construction

The 2D structure of paeoniflorin was downloaded from PubChem. After deleting repetitions, 116 potential targets relative to paeoniflorin were acquired through the TCMSP database, the TCMID and the SwissTargetPrediction database, and a total of 133 genes were identified as targets of MDD *via* the TTD, DrugBank, and GeneCards databases.

**FIGURE 3:**
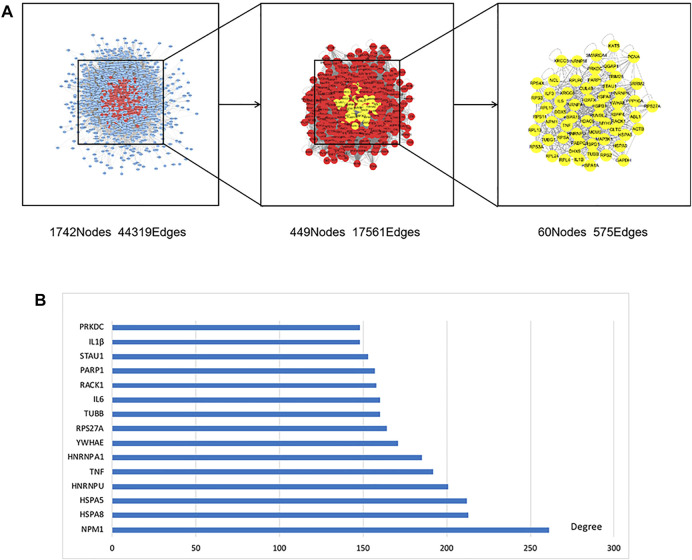
PPI network and top 15 in core targets. **(A)** 1742 nodes and 44319 edges were obtained from the intersection of paeoniflorin and MDD networks. Afterwards, the protein network with 449 nodes and 17561 edges was screened by Degree value >64. Finally, the core target protein network containing 60 nodes and 575 edges was constructed by meeting greater than median of degree, closeness, betweenness, neighborhood Connectivity and local average connectivity at the same time. **(B)** In order to further visually display the core targets, the top 15 were ranked to form a histogram.

The obtained targets in the two groups were imported separately into the bisogenet plug-in of Cytoscape 3.8.0 software in order to construct the protein-protein interaction (PPI) network. The PPI network related to potential targets of paeoniflorin consisted of 3998 nodes and 90822 edges, and the targets of MDD consisted of 3234 nodes and 72359 edges (*p* < 0.05). A total of 1742 nodes and 44319 edges were obtained from the intersection of the two networks. Afterward, the protein network with 449 nodes and 17561 edges was screened by a degree value >64. Finally, the core target protein network containing 60 nodes and 575 edges was constructed with a degree value >102, closeness >0.540410133, betweeness >204.5827132, neighborhood connectivity >108.952381, and local average connectivity >21.82089552 (Figure 3A), in which we regarded 60 nodes as core targets regarding the therapeutic effect of paeoniflorin in MDD. To visually display the core targets, the top 15 were ranked to form a histogram (Figure 3B).

### Enrichment and C-T-P-D Network Analysis

**FIGURE 4:**
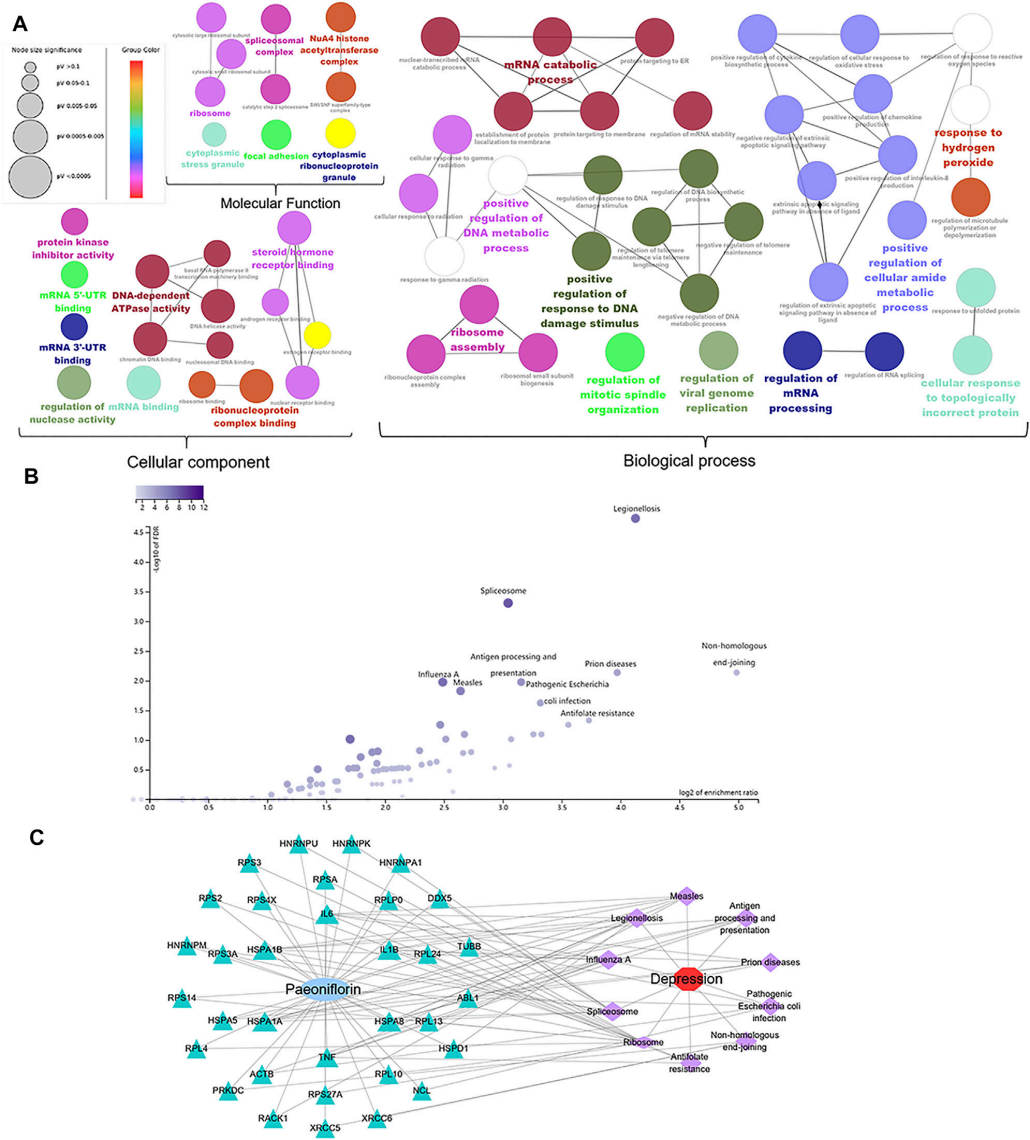
Gene ontology, KEGG enrichment and C-T-P-D network analysis. **(A)** Gene ontology analysis of 60 core genes was displayed in three modules, including biological process, cellular components, and molecular function. **(B)** KEGG signaling pathway enrichment analysis. **(C)** Accroding to the enrichment analysis of targets and pathways, we constructed the compound-target-pathway-disease network (C-T-P-D network) to visualize the relationship. Node size is proportional to the degree value.

The key targets in the therapeutic effect of paeoniflorin in MDD were uploaded to the CluoGO plug-in of Cytoscape to conduct GO analysis (Figure 4A), and carry out KEGG enrichment analysis by WEB-based GEne SeT AnaLysis Toolkit (Figure 4B). After enrichment analysis, we constructed a C-T-P-D network to visualize their relationships (Figure 4C). Notably, the main targets of paeoniflorin against MDD are NPM1, HSPA8, HSPA5, HNRNPU, and TNF. The key targets are mainly enriched in pathways related to inflammatory and immune regulation, and the activation of the innate immune inflammatory response may play a role in the pathophysiology of MDD. Interestingly, some targets are enriched in the pathway of pathogenic *Escherichia coli* (*E. coli*) infection, which suggests that paeoniflorin may be used to treat symptoms of gastrointestinal diseases. The clinical incidence of gastrointestinal diseases in patients with comorbid depression is 43.52% (Duman et al., 2016). According to the results of network pharmacology and preliminary literature research, paeoniflorin may exert an antidepressant effect by regulating cellular inflammation, synaptic function, NF-κB signaling pathway, and intestinal inflammation.

### Paeoniflorin Inhibits Neuroinflammation in LPS-Induced BV2 Cells

**FIGURE 5:**
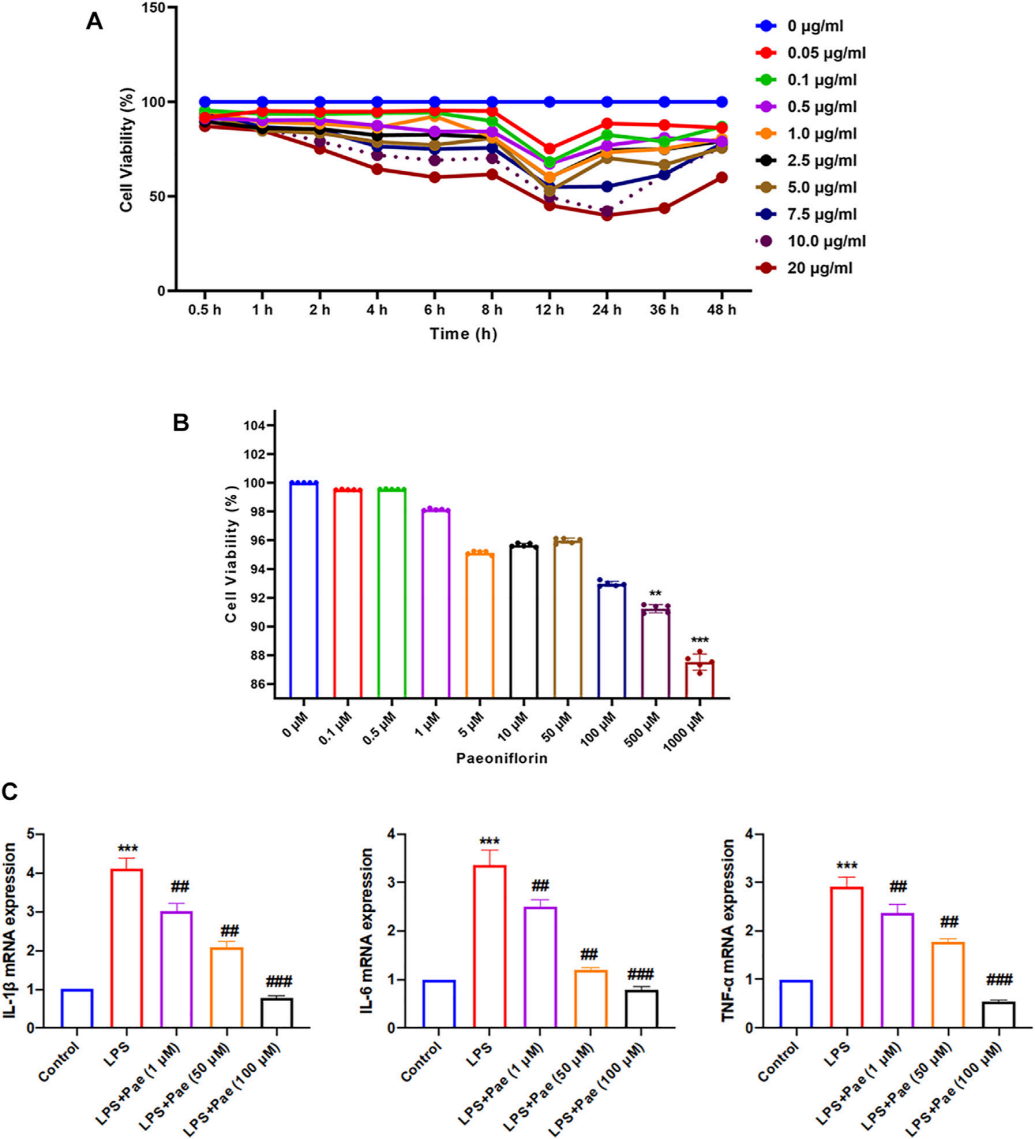
Paeoniflorin inhibits neuroinflammation in LPS-induced BV2 cells. **(A)** Effect of LPS on BV2 proliferation ability. **(B)** Effect of paeoniflorin on BV2 toxicity. **p < 0.01, ***p < 0.001 compared with 0 μM. **(C)** Effects of paeoniflorin on inflammatory cytokines secretion in LPS-induced BV2 cells. **p < 0.01, ***p < 0.001 compared with Control group. ##*p* < 0.01, ^###^ *p* < 0.001 compared with LPS group.

As shown in Figures 5A,B, different concentrations of LPS and paeoniflorin had a significant effect on the proliferation of BV2 cells over time. In the low concentration range (0.1–100 μM), the cytotoxicity of paeoniflorin on BV2 cells was not obvious. Ultimately, through repeated experiments we selected BV2 cells treated with 10 μg/ml LPS for 12 h to simulate neuroinflammation, and paeoniflorin concentrations of 1, 50, and 100 μM to intervene in LPS-induced BV2 cells. Compared with the control group, the mRNA expression levels of IL-1β, IL-6, and TNF-α in the LPS group were significantly increased (*p* < 0.001), while the LPS + Pae groups showed remarkably decreased mRNA expression levels compared to the LPS group (*p* < 0.01) (Figure 5C). The results showed that LPS can induce neuroinflammation and produce inflammatory cytokines by activating microglia, which could be inhibited by paeoniflorin.

### Paeoniflorin Improves CUMS Induced Depression-Related Behaviors

**FIGURE 6:**
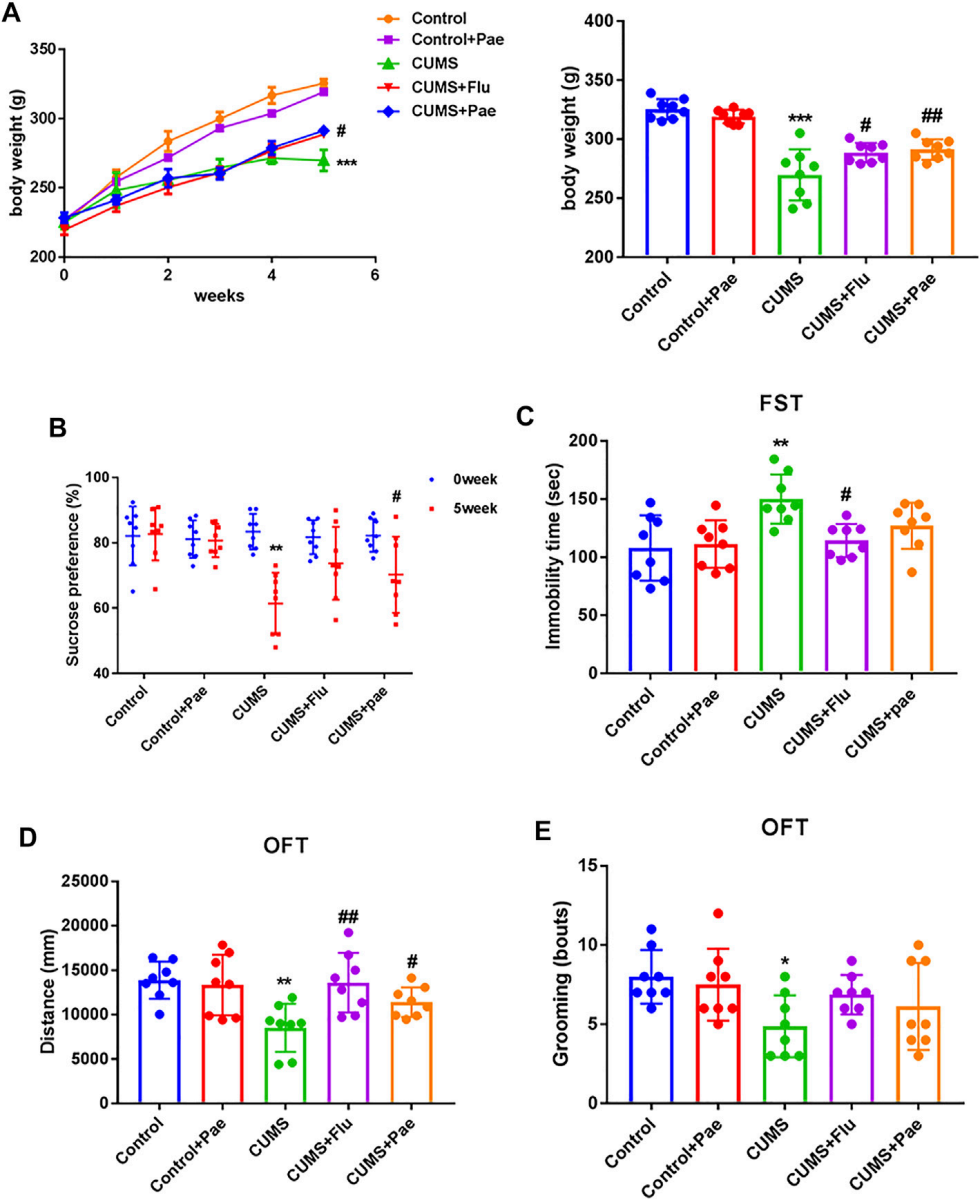
Paeoniflorin improves CUMS-induced depression-related behaviors. **(A)** Paeoniflorin can reverse the weight changes induced by CUMS exposure. ***p < 0.001 compared with control group, ^#^ *p* < 0.05, ^##^ *p* < 0.01 compared with CUMS group. Paeoniflorin can increase the sucrose consumption **(B)**, the immobile time **(C)** and the number of grooming times **(E)**, decrease the total movement distance **(D)** in CUMS-induced rats. *p < 0.05; **p < 0.01 compared with control group, ^#^ *p* < 0.05, ^##^ *p* < 0.01 compared with CUMS group. Results are presented as mean ± SD, *n* = 8.

We recorded the body weight of rats in the five groups at the first, second, third, fourth, fifth, and sixth weeks respectively in order to evaluate the effects of CUMS and paeoniflorin on the body weight of rats, and there was almost no significant difference in the body weight of the rats before the experiment, as shown in Figure 6A. After 3 weeks, the body weight of CUMS-treated rats increased more slowly than before (*p* < 0.05), indicating that CUMS may affect the regular weight growth of rats. Rats in the Control + Pae group showed no significant difference in weight changes compared with the control group (*p* > 0.05), and the surprise was that the CUMS + Pae group gained significantly more weight than the CUMS group (*p* < 0.05). These results suggest that paeoniflorin itself did not affect the weight changes in rats, and it had the same effect as fluoxetine in reversing the weight changes induced by CUMS exposure.

To clarify the function of paeoniflorin in CUMS rats, depressive-like behaviors were assessed using the SPT, FST and OFT. At the beginning of the SPT, the percentage of sucrose water consumed by the groups did not differ significantly (*p* > 0.05). As illustrated in Figures 6B–E, in the CUMS group, compared to the control group, consumption of sucrose water decreased remarkably during the SPT (*p* < 0.001), the immobile time increased during the FST (*p* < 0.01), and during the OFT, the number of grooming times and total movement distance decreased (*p* < 0.05). Thus, the CUMS model was successfully established in rats. In the CUMS + Flu groups, sucrose consumption increased in the SPT (*p* < 0.05), immobile time decreased in the FST (*p* < 0.05), and total movement distance increased in the OFT (*p* < 0.01) compared with that in the CUMS group. Interestingly, in the CUMS + Pae groups sucrose consumption and total movement distance increased in the SPT and OFT (*p* < 0.05), whereas the decrease of immobile time is not significant in the FST compared with the CUMS group. Rats that received fluoxetine had a longer swimming time and a shorter immobility time than animals that did not receive fluoxetine (Reardon, 2019). The fluoxetine treatment group had a significant difference in the FST experiment, and paeoniflorin did not show a significant statistical difference. Fortunately, we used other indicators to jointly evaluate depression like behaviors. At the same time, the Control + Pae group showed no significant difference from the control group (*p* > 0.05), which suggests that paeoniflorin itself can not affect results. These data indicate that paeoniflorin plays a role similar to fluoxetine in improving depression-related behaviors in CUMS-induced rats.

### Paeoniflorin Enhances CUMS-Induced Learning and Spatial Memory Ability by Maintaining Synaptic Function

**FIGURE 7:**
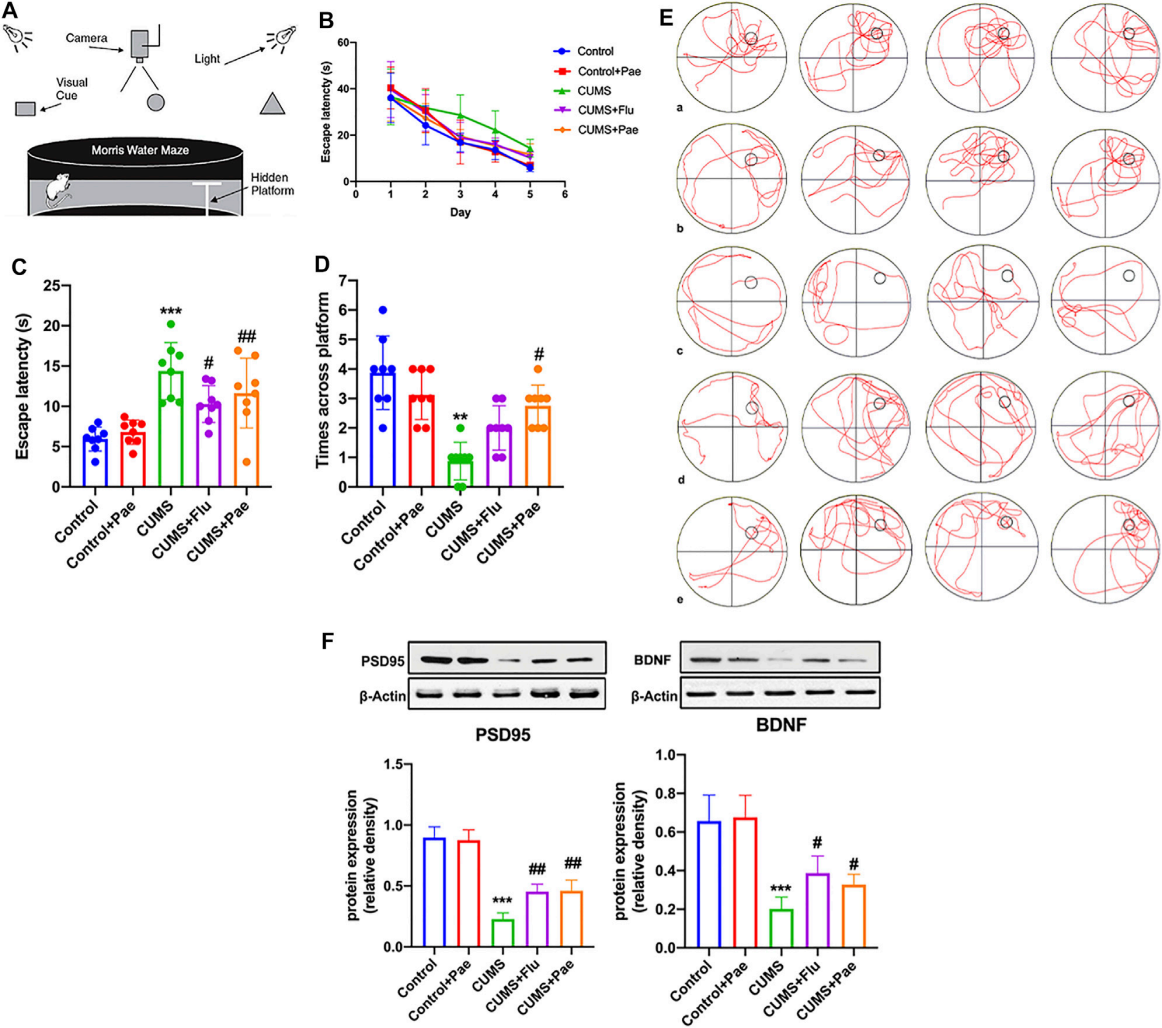
Paeoniflorin enhances CUMS-induced learning and spatial memory ability by maintaining synaptic function. **(A)** The structure and test overview of the entire maze. **(B)** The incubation time used to find the platform on day 1–5. **(C)** The latency time for rats in each group to reach the target quadrant on day 6. **(D)** The number of times rats in each group crossed the platform on day 6. **(E)** The action track of rats in each group on day 6. a: Control group, b: Control + Pae group, c: CUMS group, d: CUMS + Flu group, e: CUMS + Pae group. **(F)** Paeoniflorin can improve the expression of PSD95 and BDNF which is related with its antidepressant-like function. **p < 0.01; ***p < 0.001 compared with Control group, ^#^ *p* < 0.05, ^##^ *p* < 0.01 compared with CUMS group. Results are presented as mean ± SD, *n* = 8.

Based on network pharmacology suggesting that HSPA8 is also involved in maintaining axonal function, we tested the learning and spatial memory ability of rats in each experimental group using the MWM, and the results are shown in Figure 7A. We found that on the third day of the experiment, the latency period was significantly shorter in all the medication groups, except in the CUMS group (Figure 7B).

On the sixth day, the CUMS group required the longest time, which was significantly different from that of the control group (*p* < 0.001). Compared with the CUMS group, the latency time to reach the target quadrant after fluoxetine and paeoniflorin intervention treatment was significantly reduced (*p* < 0.05) (Figure 7C). The number of platform crossings in the CUMS group was significantly different from that of the control group (*p* < 0.01). Compared with the CUMS group, the number of platform crossings increased after treatment with paeoniflorin (*p* < 0.05) (Figure 7D). According to these data, the Control + Pae group exhibited no significant difference from the control group, which suggests that paeoniflorin itself cannot affect the ability of learning and spatial memory in rats. CUMS modeling can lead to a decline in learning and spatial memory ability in rats; however, paeoniflorin can significantly improve this phenomenon. Figure 7E shows the action tracks of the rats in each group on day 6. BDNF and PSD95, which are abundant in the postsynaptic density zone, play an important role in learning and memory and the maintenance of plasticity of synapses. Changes in their expression are reflected in pathophysiologies of the brain; therefore, we focused on changes in these two proteins in hippocampal samples (Figure 7F). Western blot analysis showed that CUMS diminished BDNF and PSD95 protein levels in the hippocampus of rats compared to control rats (*p* < 0.001); however, the protein levels increased significantly after treatment with paeoniflorin. These results indicate that paeoniflorin can improve the expression of PSD95 and BDNF, which is related to its antidepressant-like function.

### Paeoniflorin Reduces CUMS-Induced Hippocampal Inflammation by Downregulating p38MAPK/NF-κB Pathway Activation

**FIGURE 8:**
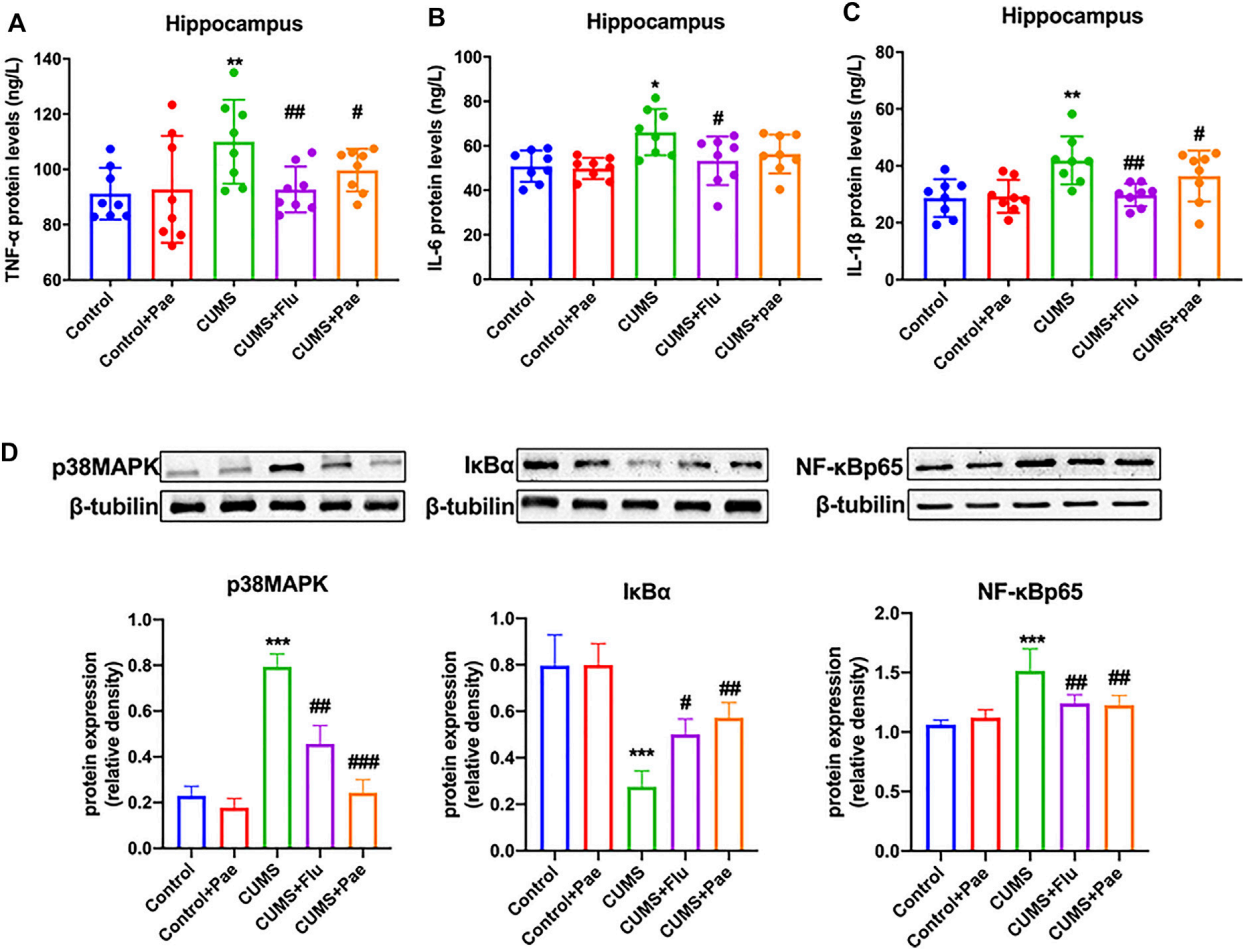
Paeoniflorin reduces CUMS-induced hippocampal inflammation by downregulating p38MAPK/NF-κB pathway activation. Paeoniflorin decreased the expression of TNF-α **(A)** and IL-1β **(C)** compared with the CUMS group, whereas no significant changes were observed in the expression of IL-6 **(B)**. **(D)** Paeoniflorin can decrease the expression of p38 MAPK and NF-κB p65, increase the expression of IκBα notably in the hippocampus of CUMS-induced rats. *p < 0.05, **p < 0.01, ***p < 0.001 compared with Control group. ^#^ *p* < 0.05, ^##^ *p* < 0.01, ^###^ *p* < 0.001 compared with CUMS group. Results are presented as mean ± SD, *n* = 8.

To investigate the anti-inflammatory effect of paeoniflorin in MDD according to network pharmacology, we performed ELISA to measure the expression levels of TNF-α, IL-6, and IL-1β in hippocampal samples from each group, as shown in Figures 8A–C. Compared with the control group, the expression levels of TNF-α, IL-6, and IL-1β in the hippocampus of the Control + Pae group were not significantly different (*p* > 0.05). The expression levels of TNF-α (*p* < 0.01), IL-6 (*p* < 0.05), and IL-1β (*p* < 0.01) in the CUMS group were significantly higher than those in the control group, which indicated that the occurrence of depression was associated with the production of inflammatory factors. The CUMS + Flu group exhibited significantly lower levels of TNF-α (*p* < 0.01), IL-6 (*p* < 0.05), and IL-1β (*p* < 0.01) compared to the CUMS group. Furthermore, the CUMS + Pae group showed decreased expression of TNF-α (*p* < 0.05) and IL-1β (*p* < 0.05) compared to the CUMS group, whereas no significant changes were observed in the expression of IL-6.

To further verify the anti-inflammatory mechanism of paeoniflorin in MDD, we analyzed the regulatory effect of paeoniflorin on the p38MAPK/NF-κB signaling pathway, according to the KEGG analysis results, as shown in Figure 8D. Compared with the control group, p38 MAPK (*p* < 0.001) and NF-κB p65 (*p* < 0.01) levels both increased; however, IκBα (*p* < 0.01) levels decreased significantly in the hippocampus of the CUMS group. The data indicated that CUMS-induced rats may activate the p38MAPK/NF-κB signaling pathway, which contributes to depression-related behaviors. At the same time, p38MAPK (*p* < 0.01) and NF-κB p65 (*p* < 0.01) expression in the hippocampus were notably reduced in comparison with the CUMS group. Hippocampal levels of p38MAPK (*p* < 0.01) and NF-κB p65 (*p* < 0.01) were significantly lower in the CUMS + Flu group than in the CUMS group. Simultaneously, hippocampal levels of IκBα (*p* < 0.05) in the CUMS + Flu group were higher than those in the CUMS group. In the hippocampus of the CUMS + Pae group, the expression of p38MAPK (*p* < 0.001) and NF-κB p65 (*p* < 0.01) were decreased, and IκBα was increased notably in comparison with the CUMS group. Western blot analyses indicated that paeoniflorin reduced CUMS-induced hippocampal inflammation by downregulating p38MAPK/NF-κB pathway activation, and it is better than fluoxetine at inhibiting the activity of inflammatory signaling pathways.

### Paeoniflorin Attenuates CUMS-Induced Intestinal Inflammation

**FIGURE 9:**
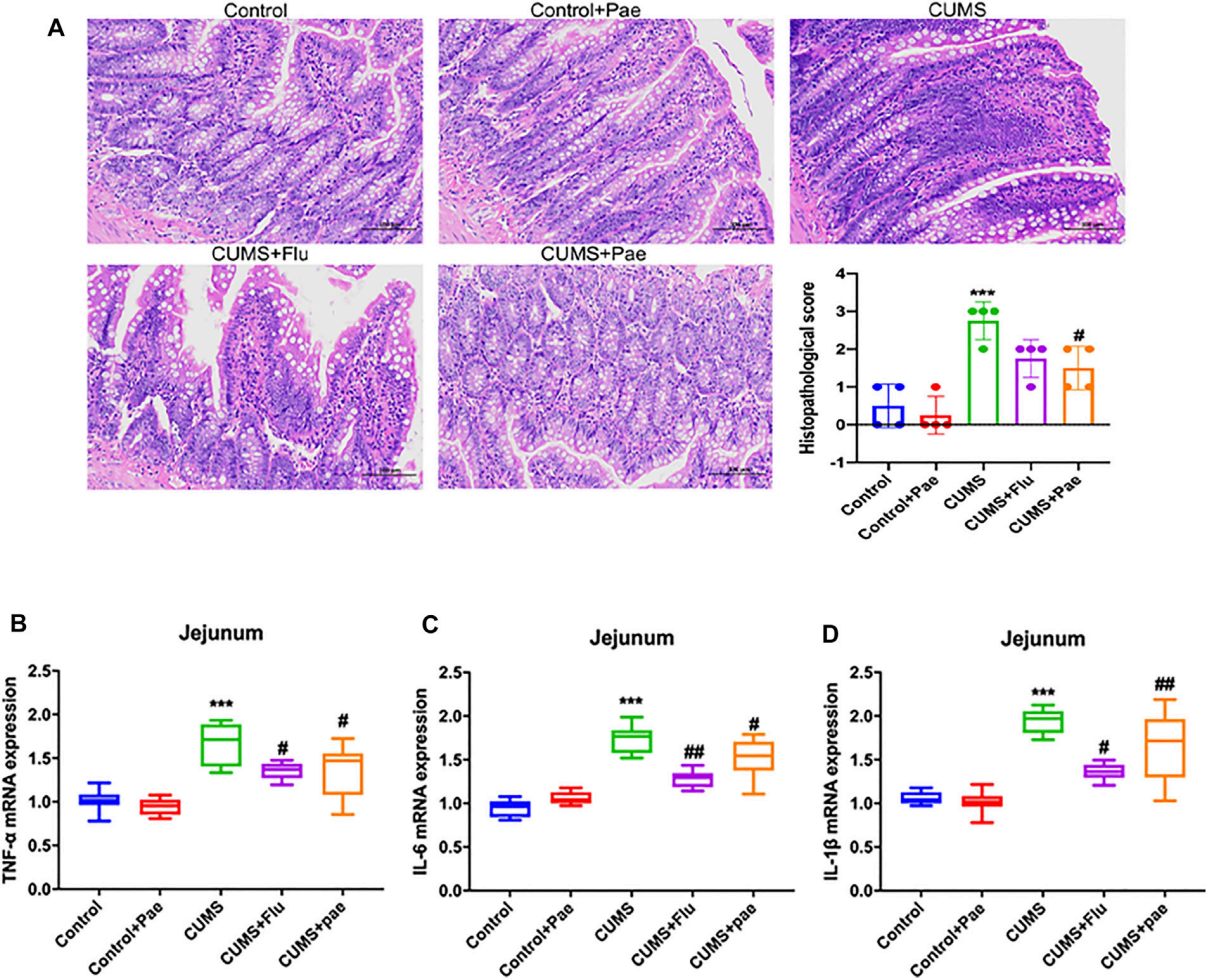
Paeoniflorin attenuates CUMS-induced intestinal inflammation. **(A)** Modeling of CUMS may lead to inflammation of the intestines in rats and paeoniflorin may reduce it. Results are presented as mean ± SD. Paeoniflorin decreases the mRNA expression of TNF-α **(B)**, IL-6 **(C)** and IL-1β **(D)** in the CUMS-induced rats. ***p < 0.001 compared with Control group, ^#^ *p* < 0.05, ^##^ *p* < 0.01 compared with CUMS group. Results are presented as “median and quartiles.”

Pathogenic *E. coli* infection is also one of the most important pathways after core target enrichment. This also reminders that paeoniflorin may treat symptoms of gastrointestinal diseases. Clinically, inflammatory bowel disease and depression have high comorbidities, with an incidence of 43.52% (Duman et al., 2016). Based on this, we evaluated the effects of paeoniflorin on CUMS-induced jejunal tissue inflammation in depressed-like rats, we randomly selected four rats in each group for jejunal pathology analysis, and the results are shown in Figure 9A. Compared to the control group, the CUMS group showed severe inflammation (*p* < 0.001), and more lymphocytic infiltration was found in the lamina propria of the intestinal villi, while no significant abnormalities were observed in the Control + Pae group (*p* > 0.05). Compared to the CUMS group, inflammatory infiltrations of jejunal tissue in the CUMS + Flu group (*p* < 0.05), and in particular in the CUMS + Pae group (*p* < 0.01) were noticeably reduced. These results suggest that the CUMS model may lead to intestinal inflammation in rats, and paeoniflorin may reduce it. For further validation, RT-qPCR was conducted to validate the effect of paeoniflorin on CUMS-induced intestinal inflammation. As shown in Figures 9B–D, no differences were found in the mRNA expression of TNF-α, IL-6, and IL-1β (*p* > 0.05) between the Control + Pae group and the Control group. The mRNA expression of TNF-α, IL-6, and IL-1β (*p* < 0.001) in the CUMS-induced rats was significantly higher than that in the control, while it was effectively reduced in the CUMS + Pae group (*p* < 0.05) compared with that in the CUMS group. The results demonstrated that paeoniflorin itself did not affect the mRNA expression of inflammatory cytokines in the jejunal samples of control rats, and both fluoxetine and paeoniflorin could decrease it in the CUMS model. Thus, paeoniflorin has a good inhibitory effect on CUMS-induced intestinal inflammation.

## Discussion

MDD is now generally recognized to be caused by changes in the central nervous system (CNS), including imbalance of neurotransmitter receptors, oxidative stress, activation of the hypothalamic-pituitary-adrenal axis (Pariante and Lightman, 2008), inflammatory cytokine imbalance (Hannestad et al., 2011), and impaired synaptic plasticity (Song et al., 2020). Antidepressants are widely used, but their adverse effects remain a difficult problem to tackle. Over the past few decades, natural products have been an important resource for pharmaceutical research and development. The development of new antidepressants can focus on Chinese herbal medicines and their active ingredients, such as the wide clinical applications of berberine (Feng et al., 2019) and puerarin (Xie et al., 2018). Network pharmacological analysis can reveal the regulatory network action of drugs and targets at the biological system level. In our study, we examined MDD-related paeoniflorin target genes through a series of bioinformatics analyses combined with subsequent experimental verification. Based on these strategies, we identified 60 genes that were related to MDD pathology and paeoniflorin treatment. The main targets of paeoniflorin against MDD are NPM1, HSPA8, HSPA5, HNRNPU, and TNF.

One of the core targets, NPM1, is involved in a variety of cellular processes, such as centrosome replication, cell proliferation, and regulation of tumor suppressor p38/TP53. Nucleophosmin, a nucleolar phosphoprotein, is the most abundant single protein identified. Co-immunoprecipitation studies suggest a physical interaction between NPM and NF-κB proteins (Dhar et al., 2004). Based on the function of NPM1, we used CUMS depression-like rats to verify the effect of paeoniflorin on the p38MAPK/NF-κB signaling pathway. The core HSPA function is to bind LPS and mediate the inflammatory response induced by LPS (Triantafilou et al., 2001). We use microglia, resident macrophages of the CNS, for verification. It plays an important role in regulating immune responses and neuronal homeostasis. Microglia are activated and secrete pro-inflammatory cytokines during inflammation. In this study, we confirmed that paeoniflorin could improve inflammatory factor secretion and reduce glial cell activation in BV2 cells induced by LPS, with good anti-inflammatory effects.

At the same time, HSPA8 can participate in the maintenance of axon function (Gaudet et al., 2011). Therefore, we used CUMS model rats to evaluate the content of paeoniflorin on postsynaptic density protein-95 and BDNF, and found that paeoniflorin can significantly increase the expression of these two proteins in the hippocampus. Glycoside can improve the depression-like behavior, learning and spatial memory ability of CUMS rats. The core target HNRNPU function is to enhance the expression of specific gene tumor necrosis factor (Yugami et al., 2007). The key pathway after enrichment also includes NF-κB. For this reason, we conducted a unified verification and found that paeoniflorin can inhibit p38MAPK, NF-κB p65 and increase IκBα. Reduce the expression of cytokines IL-1β, IL-6, TNF-α, and improve depression-like behavior in CUMS rats. Pathogenic *E. coli* infection is also a key pathway after the core target enrichment. For this reason, we detected the HE staining of the jejunum of each group of rats and the inflammatory cytokines in the jejunum. It has been proposed that paeoniflorin may exert an antidepressant effect by regulating cell inflammation, synaptic function, NF-κB signaling pathway, and intestinal inflammation.

Life stressors are the strongest proximal risk factors for depression (Kokras et al., 2021). Adverse experiences and social threats up-regulate the inflammatory response and involve parts of the immune system (Kiecolt-Glaser et al., 2015). Clinically, systemic inflammatory diseases, such as inflammatory bowel disease (Neuendorf et al., 2016), rheumatoid arthritis (Nerurkar et al., 2019), and chronic liver disease (Mullish et al., 2014), are associated with depression. The CUMS model mimics this unpredictable source of stress, causing rats to exhibit depression-like behaviors with a systemic appearance of inflammatory responses, especially in the brain and intestines. Then, we detected the inflammation levels in the hippocampus and jejunum of rats in each experimental group and found that paeoniflorin was indeed able to ameliorate the hippocampal and intestinal inflammation induced by CUMS.

**FIGURE 10:**
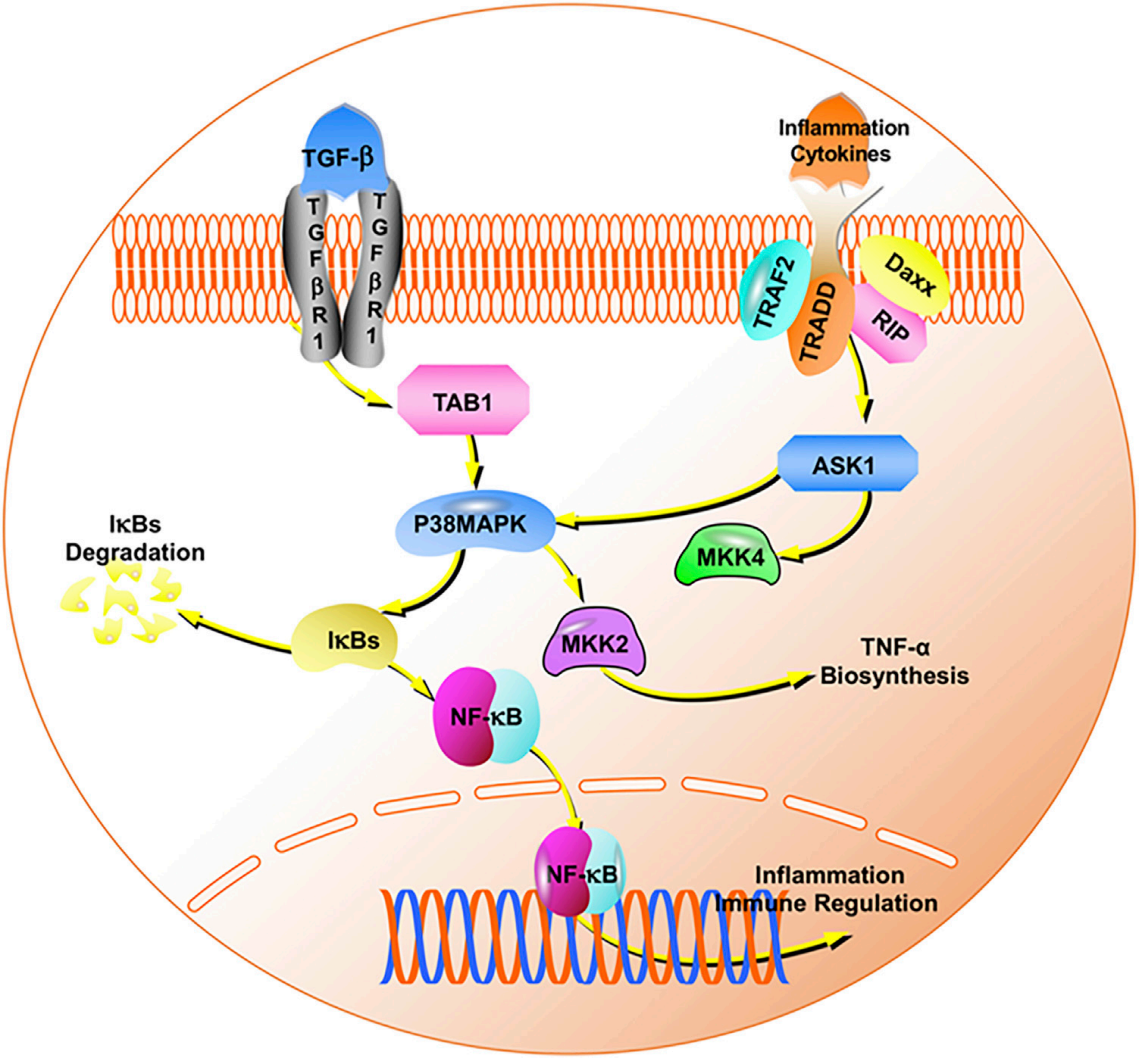
The p38MAPK/NF-κB signaling pathway.

MAPK is a group of serine-threonine protein kinases that can be activated by different extracellular stimuli, such as neurotransmitters, cytokines, and hormones. It is an indispensable transmitter in the process of signaling from the cell surface to the interior of the nucleus, including the p38MAPK transmission pathway (Cuadrado and Nebreda, 2010). Cytokines can activate it and regulate a large number of downstream transcription factors that participate in the pathophysiological processes of cell apoptosis, growth, and inflammation. Early studies found that an NF-κB inhibitory protein exists in the nucleus, which is IκBα, the first cloned IκB protein in the IκB family. Its C-terminus contains 3–8 ankyrin repeat motifs and binds to NF-κB through this motif, exposing the fixed nuclear localization signal region (NLS) of NF-κB to inhibit its activity (Hayden and Ghosh, 2004). The degradation of IκBα promotes the excessive activation of NF-κB and mediates the release of cytokines and the transcription and expression of specific genes. NF-κB is an important molecule downstream of p38MAPK. NF-κB mediates the differentiation and proliferation of various immune cells and the production of inflammatory factors (Mitchell et al., 2016). The relationship between these proteins is shown in Figure 10. The key pathways after enrichment include the NF-κB pathway. Thus, we conducted *in vivo* experimental verification and found that paeoniflorin reduced the secretion of IL-1β, IL-6, and TNF-α by inhibiting the expression of p38MAPK and NF-κB p65, while increasing the expression of IκBα.

Chronic stress also impairs the hypothalamic-pituitary-adrenal axis function and reduces neurotrophic factors such as BDNF, which can lead to impaired neuroplasticity in the hippocampus (Chiba et al., 2012). BDNF plays a central role in brain neuroplasticity, is a potent regulator of synaptic plasticity, and mediates learning and memory (Leal et al., 2014). We examined the protein expression levels of BDNF and PSD95 in the hippocampus and found that paeoniflorin reversed the CUMS-induced reduction of these two proteins, implying that paeoniflorin may improve synaptic structure (Torrisi et al., 2021) and contribute to the recovery of synaptic function (Dziedzicka-Wasylewska et al., 2021).

The interaction of neurobiological changes with the onset of neuropsychiatric disorders. Nevertheless, the crosstalk between neurobiological adaptations underlying these diseases is unclear. This bioinformatics analysis, combined with the experimental verification strategy, provides five potential paeoniflorin target genes for MDD treatment. This is the first study to use such an approach for predicting paeoniflorin treatment targets, which proves that the effect of paeoniflorin in the treatment of depression depends on multiple targets and multiple pathways, which is the advantage of paeoniflorin as an antidepressant. The limitation of our study is that *in vitro* experiments used only BV2 cells treated with LPS to simulate the MDD status. Alternative approaches using human neuronal cells, such as iPS-derived neurons, as well as multiple animal models such as the social defeat stress model, would further support our findings in the future.

## Conclusion

Patients diagnosed with depression showed elevated levels of cytokines and associated brain inflammation. Chronic inflammatory states reduce adult hippocampal neurogenesis, which in turn leads to behavioral disturbances, learning and memory deficits (Brites and Fernandes, 2015; Chesnokova et al., 2016). CUMS rats have depression-like behaviors, and related inflammation and nerve damage in the brain. P38MAPK/NF-κB signaling pathway is involved in the protective effect of paeoniflorin. The pharmacodynamic results of paeoniflorin (Figures 5, 8) showed that it had an anti-inflammatory mechanism, and at the same time, it was confirmed that it had neural memory protection effect (Figure 5). The generation of inflammation may not be confined to the brain, as the network pharmacology analysis provides clues to suggest possible gut involvement, and paeoniflorin also improved intestinal inflammation (Figure 9), thus proving the drug’s dual anti-inflammatory relations; hence, the gut, brain, and nerve protection seem to have been established. It is one of the potential mechanisms of paeoniflorin in the treatment of depression-like CUMS rats.

In our study, we combined network pharmacology and experimental verification, and it was found that paeoniflorin exerts an antidepressant effect through different targets and pathways. This confirmed the accuracy of network pharmacological prediction and further proved the feasibility of applying network pharmacology to drug analysis. Our study provides valuable information for investigating paeoniflorin in MDD and the potential mechanisms of paeoniflorin in the treatment of MDD.

## Data Availability

The original data presented in the study are included in the article/Supplementary Material, further inquiries can be directed to the corresponding authors.
